# Cytomegalovirus pneumonia in a patient with T-lymphoblastic leukemia/lymphoma after allogeneic hematopoietic stem cell transplantation: A case report

**DOI:** 10.1097/MD.0000000000014221

**Published:** 2019-01-25

**Authors:** Qiuyu Li, Kai Wang, Wei Guo, Ming Lu

**Affiliations:** a Department of Respiratory and Critical Care Medicine, Peking University Third Hospital; b Department of Clinical Laboratory, Haidian Hospital, Third Hospital of Peking University; c Department of Radiology, Peking University Third Hospital, Beijing, China.

**Keywords:** allogeneic hematopoietic stem cell transplantation (allo-HSCT), cytomegalovirus enteritis, cytomegalovirus infection, cytomegalovirus pneumonia

## Abstract

**Rationale:**

Allogeneic hematopoietic stem cell transplantation (allo-HSCT) is an important treatment for hematological malignancies. Common complications are opportunistic infections and graft-versus-host disease (GVHD). Cytomegalovirus (CMV) is one of the most common causes of opportunistic infections.

**Patient concerns:**

A 30-year-old male was diagnosed with T-cell lymphoma after persistent cough and lymphadenopathy. Fever, abdominal pain, diarrhea, rash, and dyspnea occurred after HSCT.

**Diagnosis:**

The young man developed severe CMV infection with CMV detected in the bronchoalveolar lavage fluid and gastrointestinal tract.

**Interventions:**

Intravenous ganciclovir and high-dose glucocorticoids were administered after the patient was diagnosed with CMV pneumonia and enteritis.

**Outcomes:**

After 3 weeks, the young man died from respiratory failure and infectious toxic shock caused by severe CMV infection.

**Lessons:**

Patients after HSCT should be closely monitored CMV-DNA in blood and other specimen, and treated first if necessary, so as to avoid the occurrence of severe infections such as CMV gastroenteritis and pneumonia.

## 1 Introduction

T-lymphoblastic lymphoma (T-LBL) is a lymphoblastoid tumor derived from T cells. This relatively rare type of cancer accounts for approximately 1.7% of all cases of adult non-Hodgkin lymphoma (NHL).^[1]^ According to the new WHO classification, T-LBL is incorporated into acute lymphocytic leukemia (ALL). Chemotherapy is the main treatment for achieving complete remission (CR). Patients with poor prognoses are recommended to undergo HSCT. Suitable donors may choose allogenic hematopoietic stem cell transplantation (allo-HSCT). Opportunistic infections and graft-versus-host disease (GVHD) continue to be the major causes of morbidity after HSCT. Viral infections, such as CMV and BK polyomavirus (BKV), are common sources of secondary infections. CMV can induce many pathophysiological processes, such as pneumonia, enteritis, retinitis, glomerulosclerosis, and central nervous system infection. Among them, CMV pneumonia is associated with the most significant mortality rate. This article is mainly about a patient with T-LBL who suffered from severe CMV infection after allo-HSCT.

## 2 Case report

**Figure 1:**
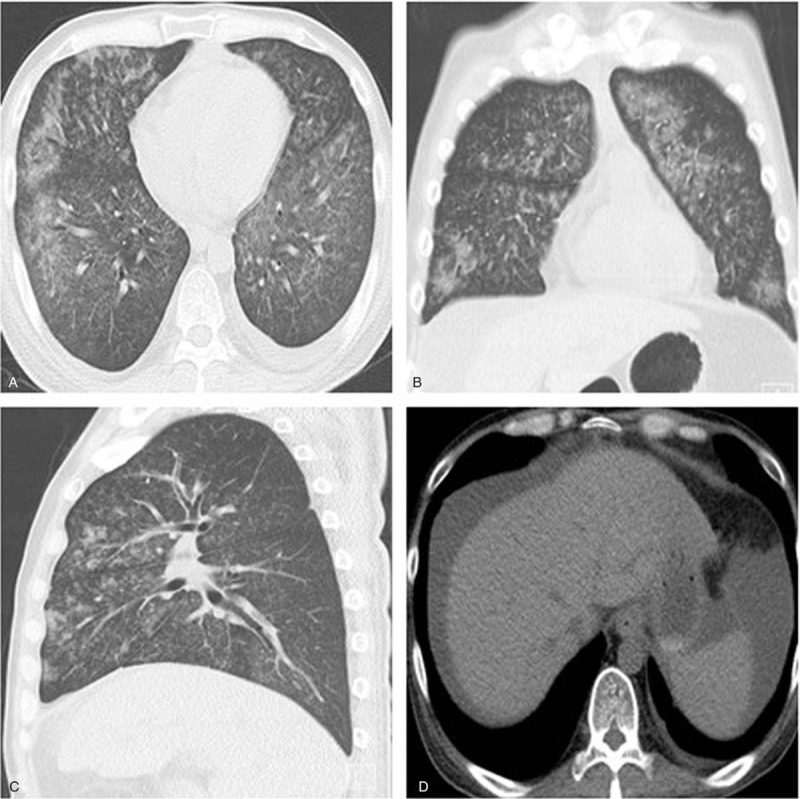
Plain axial CT scan and multiplanar reconstruction showing multiple patchy ground-glass opacities in both lungs with unclear boundaries. Changes in the air-like cavities were observed in both lungs, showing lobular or subsegmental distribution where air bronchograms were observed.

**Figure 2:**
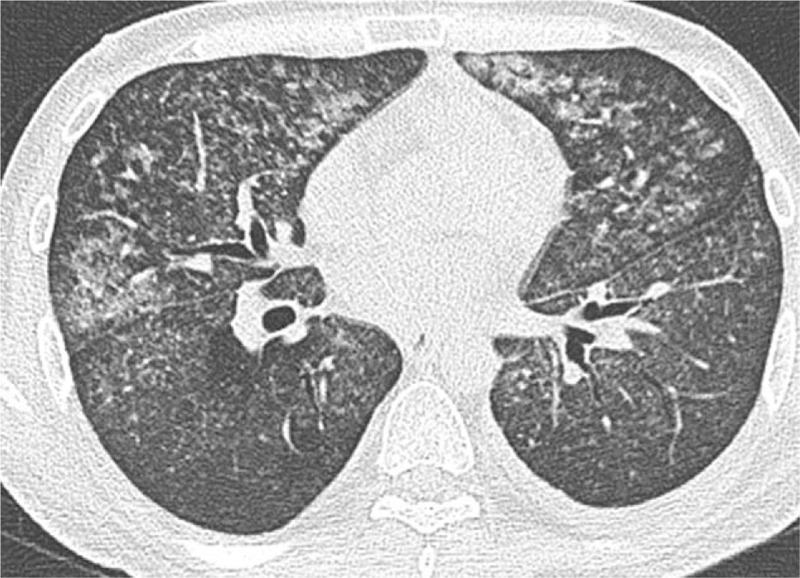
High-resolution CT scan showing diffuse multiple nodules in both lungs. Most nodules were 1 to 5 mm in diameter with smooth or irregular edges and were mostly located in the middle and inner lungs. Interlobular septal thickening, a small amount of left pleural effusion, and pleural thickening were observed.

**Figure 3:**
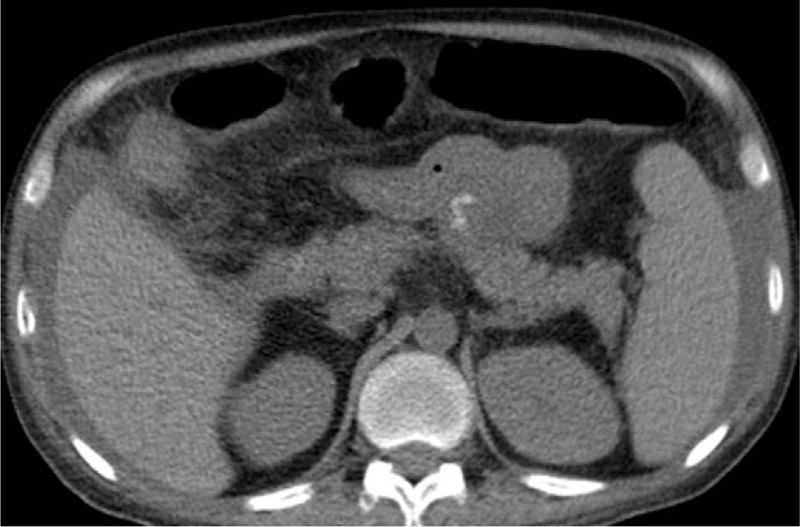
Plain abdominal CT scan showing splenomegaly and ascites.

A 30-year-old male presented with unproductive cough and multiple cervical lymphadenopathy in December 2016. The pathology of the cervical lymph node biopsy revealed T-LBL. Positron emission tomography-computed tomography (PET-CT) revealed multiple lymph node involvements in the neck, mediastinum, bilateral parasternum, and abdomen, the Ann-Arbor stage of the lymphoma reached stage III. The following treatment plan was implemented: 1 cycle of VDLP (vincristine + daunorubicin + L-asparaginase + prednisone) induction chemotherapy, followed by 2 cycles of CAM (cyclophosphamide + A-cytarabine + methotrexate) chemotherapy. After the induction chemotherapy and total body irradiation (TBI)/cyclophosphamide protocol, the patient underwent allo-HSCT with the patient's brothers as donors in July 2017. On the 12th day after transplantation, the CD34^+^ cell count was 8.33 × 10^6^/kg, the mononuclear cell (MNC) count was 10.15 × 10^8^/kg; the process was uneventful. Forty days after transplantation, the patient developed abdominal pain, diarrhea, and rash across his body. Acute grade three GVHD was considered. The symptoms were relieved after the intravenous administration of methylprednisolone, cyclosporine, and mesenchymal cells. The patient experienced fever during this period, and then the temperature returned to normal after treatment with broad-spectrum antimicrobials, including meropenem and voriconazole. Patients who showed transient BKV, CMV, and hepatitis B virus (HBV) expression levels after transplantation demonstrated improvements after antiviral treatment. The BK DNA level was checked on August 7, 2017, and the result was 4.07 × 10^7^ (copy/ml). The BK DNA level was 1.05 × 10^5^ (copy/ml). A chest CT scan showed diffuse ground-glass opacities (Figs. 1 and 2). An abdominal CT scan revealed enlargement of the spleen (Fig. 3).

The patient underwent tracheoscopy and CMV-DNA was 3 × 10^6^ in BALF and 6 × 10^3^ in blood. The patient also carried out enteroscopy at the same time and his immunohistochemical CMV antigen was positive Then, the patient was diagnosed with CMV pneumonia and enteritis and treated with intravenous ganciclovir (500 mg/d) and high-dose glucocorticoids (80 mg/d). However, the patient continued to have high fever, progressive shortness of breath, and gastrointestinal bleeding. Intubation and invasive mechanical ventilation support were performed. Unfortunately, the condition continued to deteriorate. After 3 weeks, the patient died because of severe hypoxemia and hemorrhagic shock.

Informed consent for the collection of his medical history and blood samples was obtained in compliance with the Declaration of Helsinki and approved by the local ethical committee, and the patient provided informed consent for publication of the case.

## 3 Discussion

HSCT is an effective treatment for a variety of hematological malignancies,^[1]^ but it is also known with a high failure rate. The main reasons are GVHD and infections.^[2]^ The incidence of CMV infection is high after allo-HSCT.^[2]^ This infection is mainly caused by latent CMV reactivation. Primary CMV infection is rare. Without CMV prevention or preemptive treatment, the incidence of CMV-related diseases (pneumonia, gastroenteritis, hepatitis, retinitis, and encephalitis) can be as high as 20% to 35%. Although prophylactic or preemptive therapy has been widely used, approximately 5% of these patients still develop CMV-related diseases.^[3]^

The European Conference on Infections in Leukemia (ECIL) guidelines suggest that the diagnosis of CMV infection after transplantation includes CMV, symptomatic CMV infection, and CMV target-organ disease.^[4,5]^ Before the ECIL proposal, Fishman proposed the concept of "CMV syndrome”,^[6]^ which refers to the presence of fever, fatigue, myalgia, joint pain, or myelosuppression as CMV hyperemia. CMV syndrome may be the early manifestation of CMV terminal organ disease. The diagnosis of CMV disease is based on the detection of CMV DNA or CMV antigens^[1]^ in the blood.^[5]^ In recent years, real-time quantitative PCR has become a standard method for detecting CMV DNA because of its high sensitivity and specificity.^[1]^ In recent years, some studies have suggested that there is a separate phenomenon between peripheral blood and tissue CMV specimens, that is, peripheral blood is negative while tissue specimens are positive for CMV.^[7,8]^ CMV enteritis is particularly common.^[7]^ In this case, this patient was not diagnosed with CMV pneumonia at the early stage after admission. A tissue biopsy should be collected when the peripheral blood is negative for CMV but CMV terminal organ disease is highly suspected.

Factors affecting the incidence and development of CMV infection and related diseases after HSCT include the serological status of the donor, the intensity of preconditioning, the type of donor, the HLA matching, and the application of anti-GVHD and immunosuppressive agents. Two drug-based strategies are mainly used to prevent CMV infection: nonspecific prevention and specific prevention strategies. In nonspecific prevention, all transplant patients are treated with antiviral drugs. The effect of antiviral prophylaxis before transplantation has not yet been confirmed in the literature. Treatment with nonspecific antiviral drugs after transplantation can reduce the rate of CMV reactivation, but patients may experience adverse reactions, such as drug-induced myelosuppression and secondary infection,^[9,10]^ which may also lead to drug resistance.^[10]^ Therefore, the current guidelines only recommend nonspecific prevention in high-risk CMV patients.^[4,10]^ Specific prevention is preemptive treatment for CMV, which is the best measure for preventing CMV infection. Monitoring peripheral blood for CMV after transplantation is the basis of preemptive treatment.^[4,9,10]^ A variety of guidelines recommend weekly peripheral blood CMV tests for at least 100 days after transplantation. In high-risk patients (such as those with unrelated and unmatched donors), the monitoring time should be extended to 6 to 12 months. Preemptive treatment lasts for at least 2 weeks. If the CMV DNA test result is still positive, treatment should continue. Ganciclovir is a first-line drug for preemptive treatment, but it is often stopped because of myelosuppression. Ganciclovir should be used cautiously in patients with a WBC count <0.5 × 10^9^/L or a PLT count <20 × 10^9^/L. Foscarnet and cidofovir are second-line drugs for preemptive therapy, but they can cause renal toxicity as an adverse reaction.

Although the primary therapeutic effect has been confirmed, approximately 5%^[3]^ of patients still experience CMV organ involvement. The treatment of CMV-related diseases is divided into induction therapy and maintenance therapy. However, the course of treatment remains uncertain. Induction therapy is the beginning of the treatment applied to relieve symptoms and achieve a negative peripheral blood CMV result, which usually takes approximately 3 to 4 weeks. Upon achieving a negative CMV results, treatment continues for 2 to 4 weeks. The recurrence rate of CMV disease after transplantation has rarely been reported. In this case, after receiving the first diagnosis of CMV pneumonia, the patient was given antiviral therapy according to the clinical symptoms and imaging findings.

In this case, the patient was given antiviral therapy for 6 weeks according to the clinical symptoms and imaging findings after the first diagnosis of CMV pneumonia, but CMV pneumonia recurred after treatment withdrawal. Because the peripheral blood CMV DNA result was negative throughout this process, the disease state could not be judged by the peripheral blood CMV DNA result. During the second treatment of CMV pneumonia, we obtained the ideal result based on the examination of CMV DNA in bronchoalveolar lavage fluid. Therefore, whether CMV can be detected in tissue specimens or secretions can decide the course of treatment. The first-line treatment of CMV-related diseases is the same as in second-line antiviral drug selection and preemptive treatment.^[4,10]^ Ganciclovir combined with phosphonoformic acid does not increase efficacy but often increases toxicity. Cidofovir combined with ganciclovir or foscarnet sodium can improve the curative effect.^[11]^ CMV resistance is relatively rare. However, if it exists, the second-line treatment needs to be replaced. In the early stage of treatment, some patients show a poor response to antiviral drugs, which may also be related to other autoimmune diseases, such as GVHD. The role of intravenous immunoglobulin (IVIG) in the treatment of CMV-related diseases has been controversial. However, considering the high mortality of CMV-related diseases, IVIG is still recommended in severe cases. New antiviral drugs include maribavir, letermovir, and CMX. However, their efficacy has not been confirmed in large numbers of clinical studies.^[12]^

The main mechanism of CMV-related diseases is that the low immune function after transplantation cannot effectively control CMV replication, leading to the occurrence and development of CMV-related diseases. Therefore, promoting immune recovery and reconstruction specifically for CMV after transplantation is key for controlling CMV infection. The peak incidence of CMV infection and/or reactivation is usually 1 to 3 months after transplantation. At this time, the specific treatment of CMV infection after transplantation can be achieved by reducing the use of immunosuppressive agents. However, even with immune recovery and reconstruction, there is an increased risk of GVHD. CMV adoptive cell immunotherapy includes treatment with CMV-specific cytotoxic T lymphocytes (CMV-CTLs) and donor lymphocyte infusion (DLI). Although some studies have demonstrated a good curative effect on CMV via adoptive cell immunotherapy, there are still many shortcomings, such as the increased incidence of GVHD, the treatment not being available in time, and the treatment being expensive. Therefore, the international guidelines have not yet recommended a first- or second-line scheme^[4,10]^ of adoptive cell immunotherapy. Sources include second- and unrelated third-party donors. CMV-CTLs can be isolated directly from peripheral blood or obtained by in vitro antigen stimulation amplification.^[12]^ Leen et al^[13]^ reported that the effective rate of CMV-CTLs in the treatment of CMV-related diseases was 73.9%. The effective rate of CMV-CTLs at the Peking University People's Hospital for the treatment of CMV-related diseases resistant to ganciclovir and foscarnet sodium was 57%.^[14]^ DLI is a very simple and effective adoptive immunotherapy, but it increases the risk of GVHD and is limited in clinical application. A clinical study on refractory CMV infection was carried out in the department of hematology of Nan-fang Hospital of China. If the patient had no GVHD signs and the incidence of infection was 3 months after transplantation, treatment with DLI and mesenchymal stem cells (MSCs) (Clinical Trial Registration No. NCT02083731) was added on the basis of immunosuppressant reduction. The preliminary results of small samples suggested that the treatment might be effective without increasing the risk of GVHD.

CMV pneumonia is the most common end-organ disease of CMV infection after HSCT. In recent years, CMV pneumonia after aGVHD has attracted much attention^[7]^ because diagnosing and treating these patients is very difficult. CMV enteritis usually occurs after the treatment aGVHD. The main clinical manifestation is that the intestinal GVHD is not ideally controlled or becomes worse with treatment. For patients with this condition, the possibility of CMV enteritis should be highly suspected. Colonoscopy biopsy and CMV detection in intestinal exfoliated mucosa should be carried out as soon as possible. Because of virus isolation in peripheral blood and tissue samples, patients negative for CMV DNA in the peripheral blood should also undergo testing for CMV in tissue samples as soon as possible. For such patients, clinicians should try to reduce the dose of immunosuppressive agents and administer antiviral drugs at the same time. It is worth noting that these patients usually have hematopenia and other complications and cannot tolerate antiviral drugs, making adoptive immunotherapy a potentially better choice.

CMV infection has been proven to be a risk factor for GVHD. Both domestic and foreign studies have found that CMV pneumonia may be a risk factor for bronchial obliteration (BO). The mechanism may be that the inflammatory damage to the lung parenchyma and small airways in CMV pneumonia causes the formation of pulmonary interstitial scars and fibrosis; in addition, CMV infection causes a T-cell immune response and ultimately leads to the occurrence and development of BO.^[15,16]^

In conclusion, CMV infection is a common complication after HSCT and is closely related to GVHD, implants and other infections. CMV terminal organ disease still has a high mortality rate. With the extensive application of unrelated- and haploid-donor HSCT, the incidence of CMV infection seems to be increasing. Effective CMV management after transplantation can reduce diseases related to CMV infection and organ involvement.

## Author contributions

**Conceptualization:** Qiu-yu LI.

**Data curation:** Qiu-yu LI.

**Formal analysis:** Qiu-yu LI, Kai WANG.

**Funding acquisition:** Qiu-yu LI, Kai WANG.

**Resources:** Wei Guo.

**Writing – original draft:** Qiu-yu LI.

**Writing – review & editing:** Qiu-yu LI, Ming Lu.
